# Combined DNA-PK and PARP Inhibition as a Therapeutic Strategy in BRCA-Mutated Prostate Cancer: An in Vitro Pilot Study

**DOI:** 10.1177/15330338251394948

**Published:** 2025-11-14

**Authors:** Thomas Paul Scherer, Souzan Salemi, Valentin Baumgartner, Dominik Enderlin, Alekhya Mazumdar, Daniel Eberli

**Affiliations:** 1 Department of Urology, 27243 University Hospital Zurich (USZ), University of Zurich (UZH), Zurich, Switzerland

**Keywords:** talazoparib, nedisertib, prostate cancer, *in vitro*

## Abstract

**Introduction:**

DNA double-strand breaks (DSBs) are repaired via homologous recombination (HR) or the more error-prone non-homologous end joining (NHEJ). *breast cancer gene 1 (BRCA1)* and *breast cancer gene 2 (BRCA2)* are key genes in HR, and their mutations are associated with aggressive prostate cancer (PCa). While PARP inhibitors (PARPi) improve survival in BRCA-mutated PCa, their efficacy in late-stage disease is limited and often accompanied by serious side effects. This study aims to develop an in vitro model of BRCA-mutated PCa and evaluate the therapeutic potential of DNA-dependent protein kinase (DNA-PK) inhibitors that target the NHEJ pathway.

**Methods:**

The genes *BRCA1* and *BRCA2* were targeted for knockout (KO) in lymphnode cancer of the prostate (cell line) [LNCaP] using clustered regularly interspaced short palindromic repeats (CRISPR)-Cas9 technology. The KO were assessed via Western blot analysis. Scramble LNCaP, *BRCA1* KO, and *BRCA2* KO cells were treated with the PARPi talazoparib, the DNA-PK inhibitor nedisertib and their combination. The impact on cell proliferation was evaluated using the CellTiter-Glo assay and synergy of the treatments was analyzed with SynergyFinder. Cytotoxic effects were measured by flow cytometry using an Annexin V-fluorescein isothiocyanate (FITC) apoptosis detection kit. The presence of DSB was quantified using immunofluorescence.

**Results:**

BRCA2 and BRCA1 protein expression were successfully downregulated in the knockout (KO) cell lines. After two days of treatment with talazoparib and/or nedisertib, a significant decrease in cell proliferation was observed. Additive effects of the combination treatment were detected exclusively in the BRCA KO cells. These cells also exhibited significantly higher rates of necrosis after treatment compared to scramble cells and, DNA DSB were significantly more prevalent in the BRCA KO cells. Additionally, BRCA1/2 loss is inversely correlated with DNA-PK, with knockout leading to increased DNA-PK expression to support NHEJ.

**Conclusion:**

BRCA knockout LNCaP models were established, exhibiting increased DNA-PK activity and indicating greater susceptibility to DNA-PK inhibition. Therapeutically targeting NHEJ presents a promising approach in treating BRCA-mutated PCa. Further *in vivo* investigations are required to assess the tolerability of this drug combination.

## Introduction

The development of aggressive prostate cancer (PCa) is frequently attributed to hereditary factors that compromise DNA damage repair systems.^1,2^ These genetic alterations specifically contribute to an elevated risk of developing PCa at a younger age, and are closely associated with more aggressive disease features.

The *Breast Cancer Gene 1 (BRCA1)* and *breast cancer gene 2 (BRCA2)* genes are among the most important predisposing germline alterations.^1,3^ The homologous recombination (HR) repair pathway heavily depends on *BRCA1* and *BRCA2* genes for fixing DNA double-strand breaks (DSBs).^4^ By repairing DSBs, these genes are essential for preserving genomic stability and controlling the cell cycle. Patients carrying *BRCA* mutations, therefore, exhibit severely impaired HR repair capacity, which forces reliance on error-prone DNA repair pathways such as non-homologous end joining (NHEJ). HR functions mainly in the S/G2 phases when a sister chromatid is available, while NHEJ is active throughout most of the cell cycle, particularly in G1.^5^ The shift to NHEJ leads to a high mutational burden and genomic instability, contributing to the development of more aggressive prostate cancer. Clinically, this is reflected in higher Gleason scores, more advanced tumor stages at diagnosis, increased risk of lymph node involvement and distant metastasis, and ultimately, poorer prognosis and reduced overall survival (OS).^6–10^ Furthermore, somatic mutations of HR repair genes are frequently found in metastatic castration-resistant PCa.^11^

Poly (ADP-Ribose) Polymerase (PARP) enzymes, particularly PARP1, play crucial roles in the repair of DNA single-strand breaks (SSBs) through the base excision repair^12^ PARP inhibitors (PARPi) act by blocking PARP's catalytic activity to prevent SSB repair and by inducing “PARP trapping,” where PARP enzymes become stably bound to DNA at damage sites.^13^ These lesions can collapse replication forks during S phase, forming DSBs. While functional HR allows normal cells to repair DSBs, HR-deficient cancer cells (eg, with *BRCA1/2* mutations) cannot, resulting in genomic instability, cell cycle arrest, and apoptotic death.

This synthetic lethal interaction forms the basis for the clinical efficacy of PARPi, which are used for the treatment of metastatic castration-resistant PCa in patients with mutations in *BRCA1* and *BRCA2*.^14,15^ Although PARPi are initially effective, their long-term therapeutic effectiveness is limited since resistance to them often arises within months of starting medication.^16^

To address resistance to PARPi, we propose that concurrent inhibition of the NHEJ pathway may offer a rational therapeutic strategy. DNA-dependent protein kinase (DNA-PK), the catalytic subunit of the DNA-PK holoenzyme, plays a central role in classical NHEJ and is essential for its function.^17^ Inhibiting DNA-PK kinase activity disrupts NHEJ, thereby blocking the repair of DSBs through this pathway. In HR-deficient cells, which depend heavily on NHEJ for survival, this disruption may lead to a critical accumulation of unrepaired DSBs and ultimately induce cell death. Therefore, we hypothesize that combining PARP and DNA-PK inhibitors could synergistically eliminate *BRCA1/2*-deficient cancer cells. This study aimed to assess the effect of impairing the alternative DSB repair, the NHEJ pathway, using Inhibitors of DNA-PK in BRCA mutated PCa.

## Material & Methods

### cBioPortal Database and Bioinformatics Analysis

Mutation, structural variant, and copy number alterations in the *BRCA1* and *BRCA2* genes were analyzed using data obtained from cBioPortal for Cancer Genomics (https://www.cbioportal.org/, accessed on September 23, 2025).^18–20^ A total of 12 361 tumor samples from 11 893 prostate cancer patients across 18 comprehensive studies were included in the analysis.^21–40^ Raw data on mutation counts and alteration frequencies were downloaded from cBioPortal and processed using RStudio (version 2024.12.1 + 563 for macOS).

Patient-level mean alteration frequencies for *BRCA1* and *BRCA2* mutations and deep deletions were computed using a patient-weighted average rather than a study-weighted average, and results were visualized using RStudio. Clinical data from patients were similarly retrieved from cBioPortal and used for survival analysis. Patients with any *BRCA1* or *BRCA2* alteration (mutation or deep deletion) were grouped and compared against patients with wild-type (unaltered) *BRCA1* and *BRCA2* genes. Kaplan-Meier survival curves were generated using RStudio. To ensure statistical relevance, survival analysis was limited to groups containing a minimum of five patients. Hence, overall survival was analyzed from 0 to 80 months post-diagnosis.

### Pharmacological Compounds

Talazoparib and nedisertib were both obtained from Selleck Chemicals (Houston, TX, USA).

### Generation of CRISPR/Cas9-Mediated Knockout Cells

clustered regularly interspaced short palindromic repeats (CRISPR)/Cas9 Knockout cells were generated as described by Mazumdar et al.^41^ Briefly, single-guide RNAs (sgRNAs) against BRCA1 and BRCA2 genes were designed with the online tool CHOPCHOP v3 (https://chopchop.cbu.uib.no;^42^). The top 2-3 predicted sgRNAs for highly specific target sites were selected and the respective sequences are listed in Supplementary Table 1. A non-targeting control single-guide RNA (sgRNA) (sgScramble) was chosen from the same tool. Oligonucleotides specific for the target sites were synthesized with BsmBI restriction site overhangs by Microsynth (Balgach, Switzerland) and then annealed and cloned into the lentiCRISPRv2 transfer plasmid, (Addgene plasmid # 52961).^43^ Lentiviral particles were produced by the co-transfection of the lentiCRISPRv2 plasmid, containing the respective gRNA-sequence, with the packaging plasmids pCMV-VSV-G (Addgene plasmid # 8454) and psPAX2 (Addgene plasmid # 12260) into human embryonic kidney (HEK)-293T cells. 1 mg/ml of Transfection Grade Linear Polyethylenimine Hydrochloride (PEI MAX^TM^) (Polysciences Europe GmBH, Hirschberg an der Bergstrasse, Germany) was used as the transfection reagent according to the manufacturer's instructions. For transduction, LNCaP cells were incubated for 24 h with supernatant containing the viral particles and 8 µg/mL polybrene (Merck Millipore, Molsheim, France). Subsequently, the cells were selected with 2.5 µg/mL puromycin (Thermo Fisher Scientific, Paisley, UK) for 7-14 days. The efficiency of the knockouts was tested with Automated western blotting (WES; ProteinSimple, San Jose, CA, USA).

### Cell Culture

The prostate cancer cell line LNCaP (RRID: CRL-1740) was obtained from LGC Standards GmbH (Wesel, Germany). HEK293T/17 (RRID: CRL-11268) cells were kindly provided by A. Fahrner and E. Luca (Division of Endocrinology, Diabetes, and Clinical Nutrition, University Hospital Zurich, 8091, Zurich, Switzerland). LNCaP cells were cultured in RPMI medium (Thermo Fisher Scientific, Waltham, MA, USA) supplemented with 10% fetal bovine serum (FBS, Sigma-Aldrich, St. Louis, MO, USA) and 1% penicillin/streptomycin (P/S, Gibco). HEK293T/17 cells were cultured in Dulbecco's Modified Eagle Medium (DMEM)/F-12 GlutaMAX medium (Thermo Fisher Scientific, Waltham, MA, USA) supplemented with 10% FBS and 1% P/S. The cultures were maintained at 37 °C in a humidified atmosphere with 5% CO_2_.

### Cell Proliferation Assay

Cell proliferative capacity was assessed using the CellTiter-Glo 2.0 assay (G9241, Promega, Madison, WI, USA). Cells were seeded in 96-well plates (TPP) at a density of 5000 cells/well and cultured for one day. On the following day, cells were treated with talazoparib and/or nedisertib for two days. Subsequently, 100 μL of a 1:2 mix of CellTiter-Glo and culture medium was added to the wells. Cells were lysed on an orbital shaker for 2 min, and 50 μL of the lysate was transferred to white 96-well plates (Cellstar). Luminescence was recorded using a Cytation 5 imaging reader (BioTek, Winooski, VT, USA).

### Flow Cytometry Apoptosis/Necrosis

Cytotoxicity was assessed in PCa cell lines by flow cytometry using the Annexin V-fluorescein isothiocyanate (FITC) apoptosis detection kit (ab14085, Abcam, Cambridge, UK) according to the manufacturer's protocol. Briefly, 200 µL of Annexin-binding buffer was added to the cell pellet in FACS tubes (BD) and placed on ice. 2 µL of Annexin V and 2 µL of propidium iodide (PI) were added to the cell suspension and incubated for 5 min at room temperature (RT) in the dark. Data were acquired using LSRFortessa (BD, San Jose, CA, USA) and analyzed by FlowJo (V10).

### Immunostaining of Double-Strand Breaks

Cells were cultured on 4-well chamber slides (Thermo Fisher Scientific, Waltham,MA, USA). Cells were fixed with ice-cold paraformaldehyde for 10 min and permeabilized with 0.2% Triton X-100 in phosphate-buffered saline (PBS) for 10 min. 3% Bovine serum albumin (BSA) + 1% normal goat serum in PBS was used to block unspecific antibody binding (1 h at RT). The primary antibody Mouse anti TP53BP1 (*#*NBP2-25028*,* 1:200*,* Novus Biologicals, Zug, Switzerland) was diluted and incubated at 4 °C overnight. The secondary antibody and DAPI were diluted and incubated for 1 h, at RT: goat anti-mouse cyanine 3 (Cy3) IgG (AP124C, 1:700, Sigma-Aldrich, St. Louis, MO, USA); DAPI (4′,6-diamidino-2-phenylindole, 1:200, Thermo Fisher Scientific, Waltham, MA, USA). Cells were mounted with coverslips using a DAKO mounting medium (Agilent Technologies, Santa Clara, CA, USA). Immunostained cells were imaged using a Leica DMi8 microscope (Leica, Wetzlar, Germany).

### Simple Western Analysis

Cells were lysed in ice-cold lysis buffer containing 50 mM Tris-HCl (pH 7.4), 150mM NaCl, 10% glycerol, 1% Triton X-100, 2 mM ethylenediaminetetraacetic acid (EDTA), 10 mM sodium pyrophosphate, 50 mM sodium fluoride, and 200 µM sodium orthovanadate. A protease inhibitor cocktail (11697498001, Roche AG, Switzerland) was added at a 1:100 dilution immediately before use. Total protein concentrations were determined using the Pierce™ bicinchoninic acid (BCA) Protein Assay Kit (A55864, Thermo Fisher Scientific, Waltham, MA, USA) according to the manufacturer's instructions. All samples were normalized to a final protein concentration of 0.8 mg/mL for WES analysis. Capillary-based immunodetection was performed using the Simple WES system with the 66-440 kDa cartridge kit (SM-W008, Bio-Techne, Minneapolis, MN, USA), following the manufacturer's protocol. Primary antibodies were used at a 1:100 dilution and included mouse anti-Vinculin (MAB6896, Bio-Techne, Minneapolis, MN, USA), rabbit anti- BRCA2 (10741S, Cell Signaling Technology, Danvers, MA, USA), mouse anti-BRCA1 (NB100-598, Bio-Techne, Minneapolis, MN, USA), and rabbit anti- DNA PKcs (ab32566, Abcam, Cambridge, UK). Protein expression was quantified based on molecular weight and chemiluminescent signal intensity (height). Data analysis was conducted using Compass software (ProteinSimple, Version 5.0.1), with relative expression levels normalized to Vinculin.

### Statistical Analysis

Statistical analyses were conducted using one-way analysis of variance (ANOVA) with Bonferroni's post-hoc test in GraphPad Prism (version 9.5.1, GraphPad Software, Inc., La Jolla, CA, USA) as well as in RStudio /version 2024.12.1 + 563 for macOS). p-values < 0.05 were considered statistically significant. Drug synergy was assessed using the Bliss independence model implemented in the SynergyFinder 3.0 WebApp.^44^ Data are presented as means ± standard error of the mean (SEM).

### Ethical Considerations

This study was conducted exclusively using in-vitro experimental models. No human participants or animal subjects were involved. Hence, approval from the Ethics Committee was not required.

## Results

### Widespread Prevalence of BRCA1 and BRCA2 Mutations and Deep Deletions is Associated with a Worse Prognosis in Patients

**Figure 1.**
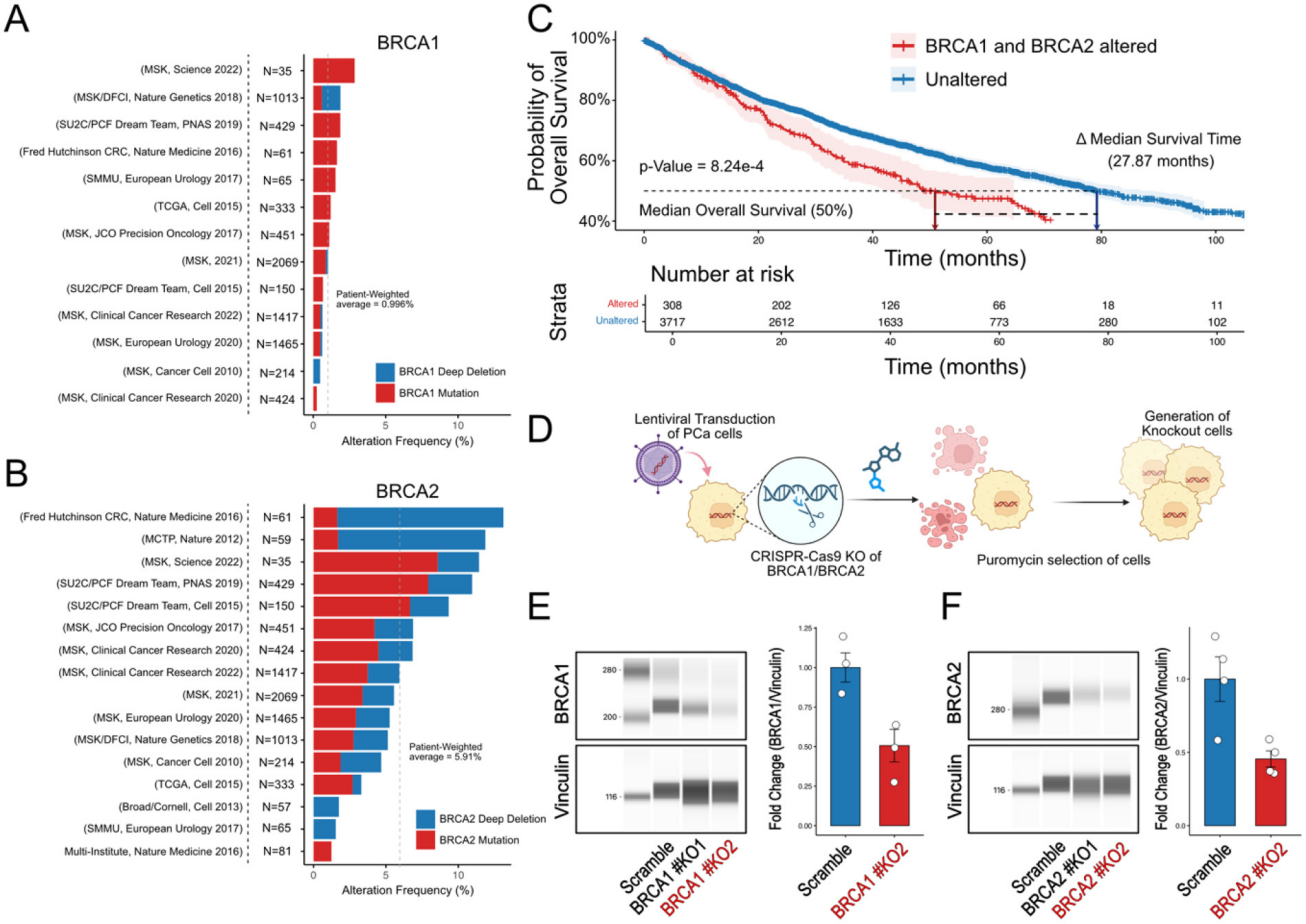
BRCA1/2 Mutations and Deep Deletions Link to Poor Outcomes. (A-B) Frequency of BRCA1 (A) and BRCA2 (B) Alterations, Including Mutations (red) and Deep Deletions (Blue), Across the cBioPortal for Cancer Genomics Database for Prostate Cancer. The Patient-Weighted Average Alteration Frequency is Indicated by a Dashed Vertical Line. (C) Kaplan–Meier Analysis of Overall Survival Comparing Patients with BRCA1/2 Alterations (Red) to Those Without (Blue), Demonstrating Significantly Reduced Survival in the Altered Group. Δ Median Survival = 27.89 Months. n = 308 in BRCA1/2 Altered Group; n = 3717 in the Unaltered Group. (D) Schematic of CRISPR-Cas9-Mediated Knockout (KO) of BRCA1 or BRCA2 in Prostate Cancer Cell Lines LNCaP. (E) Representative Virtual Lanes of Simple Western Validation of BRCA1 and BRCA2 Knockout Efficiency. (F) Representative Lane View and Densitometric Quantification Normalized to Vinculin are Shown. Graphs Represent Mean ± SEM from Three-Four Independent Experiments.

Genomic and clinical data were reanalyzed from 8676 tumor samples across 8444 patients using publicly available datasets from cBioPortal. *BRCA1* alterations were occurring in 1.00% of patients, based on a patient-weighted average across all studies (Figure 1A). In contrast, *BRCA2* alterations were substantially more frequent and present in 5.91% of the cohort (Figure 1B).

Patients with *BRCA1/2* alterations showed a significantly shorter overall survival, with a reduction of 27.89 months in median survival compared to those without alterations, at the time point corresponding to 50% overall mortality within the cohort (Figure 1C).

To model homologous recombination deficiency and evaluate responses to PARP and DNA-PK inhibition, *BRCA1* and *BRCA2* were individually knocked out in LNCaP cells using CRISPR-Cas9 gene editing (Figure 1D). Two independent guide RNAs (gRNAs) were designed per gene. Protein expression analysis via Simple Western (WES) demonstrated that gRNA #2 for both *BRCA1* (BRCA1#KO2) and *BRCA2* (BRCA2#KO2) resulted in superior knockout efficiency compared to gRNA #1 (Figure 1E; Supplementary Figure 1). Therefore, these clones were selected and used for all subsequent functional assays.

### Combined PARP and DNA-PK Inhibition Synergistically Reduces Cell Viability

**Figure 2.**
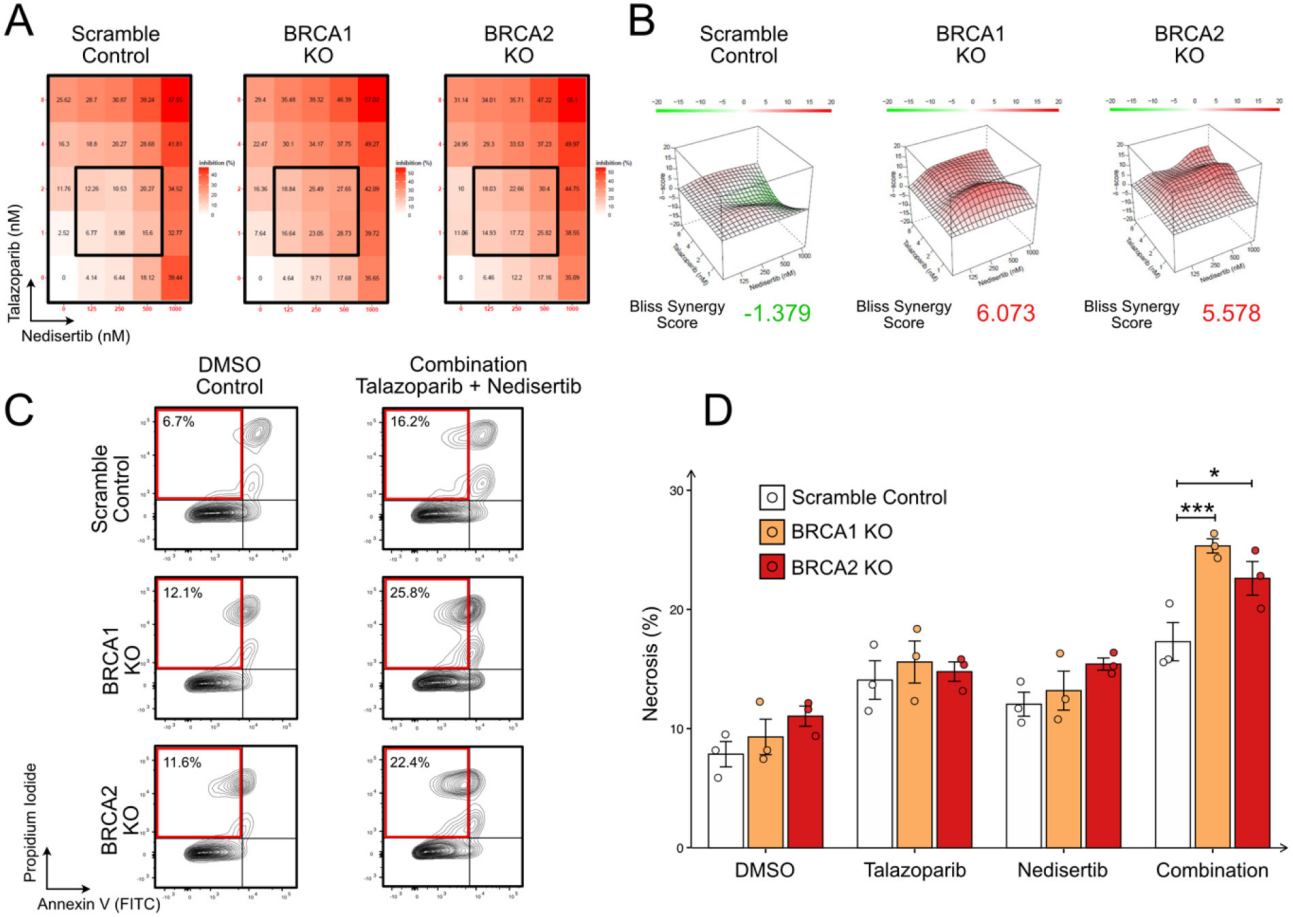
Co-Targeting PARP and DNA-PK Induces Necrosis. (A) Cell Proliferation Analysis of LNCaP Cells Treated with PARP Inhibitor (Talazoparib) and DNA-PK Inhibitor (Nedisertib) at Varying Concentrations, Measured by CellTiter-Glo 2.0. (B) Synergy Analysis of Talazoparib and Nedisertib Combinations Using SynergyFinder Showing Additive Effects of Combined PARP and DNA-PK Inhibitor Treatment in BRCA 1/2 Knockout LNCaP Cells After 48 h of Treatment. (C) Representative Flow Cytometry Contour Plots of Cell Death Analysis Using Using AnnexinV-PI Apoptosis Detection kit via Flowcytometry. Cells Were Treated with 8 nM Talazoparib, 1000 nM Nedisertib for 48 h. the red box Indicates the Quadrant Showing Necrosis (Negative for AnnexinV Staining and Positive for PI Staining). (D) Quantification of the Necrosis from AnnexinV-PI Staining from Three Independent Experiments. Graphs Represent Mean ± SEM. p-Values: p < 0.05 (*), p < 0.001 (***).

To assess the dependencies of scramble, BRCA1 KO and BRCA2 KO LNCaP cells on the repair mechanism of DSB, we examined the effect of talazoparib and nedisertib on cell proliferation. Cell viability was assessed for each drug treatment of 48 h at various dosages. Doses of 8 nM and 16 nM of talazoparib dosages demonstrated significant lower cell viability in BRCA KO LNCaP cells (Supplementary Figure 2). The dosages of 1 nM, 2 nM, 4 nM and 8 nM of talazoparib and 125 nM, 250 nM, 500 nM and 1000 nM of nedisertib were selected as concentrations for the synergy analysis as the maximum concentrations led to a growth inhibition of 20% (Figure 2A). Additive effects of the treatment of talazoparib and nedisertib were only found in the KO of BRCA 1 (Bliss synergy score 6.073) and BRCA 2 (Bliss synergy score 5.578), whereas the scramble variant did not show additive effects (Bliss synergy score −1.379) (Figure 2B).

### PARP and DNA-PK co-Inhibition Triggers Cell Death via Necrosis

To assess the cytotoxic effects of PARP inhibition, DNA-PK inhibition, and their combination, LNCaP scramble, BRCA1 KO, and BRCA2 KO cells were treated for 48 h with 8 nM talazoparib, 1000 nM nedisertib, or the combination. Annexin V/PI staining was performed to determine the mode of cell death (Figure 2C). No significant increase in Annexin V staining was observed across treatments, suggesting that apoptosis was not the predominant mechanism of cell death. In contrast, a significant increase in necrosis was detected in BRCA1 KO and BRCA2 KO cells relative to scramble controls (Figure 2D).

### Co-targeting PARP and DNA-PK Induces DSB Accumulation in BRCA1/2-Deficient Cells

**Figure 3.**
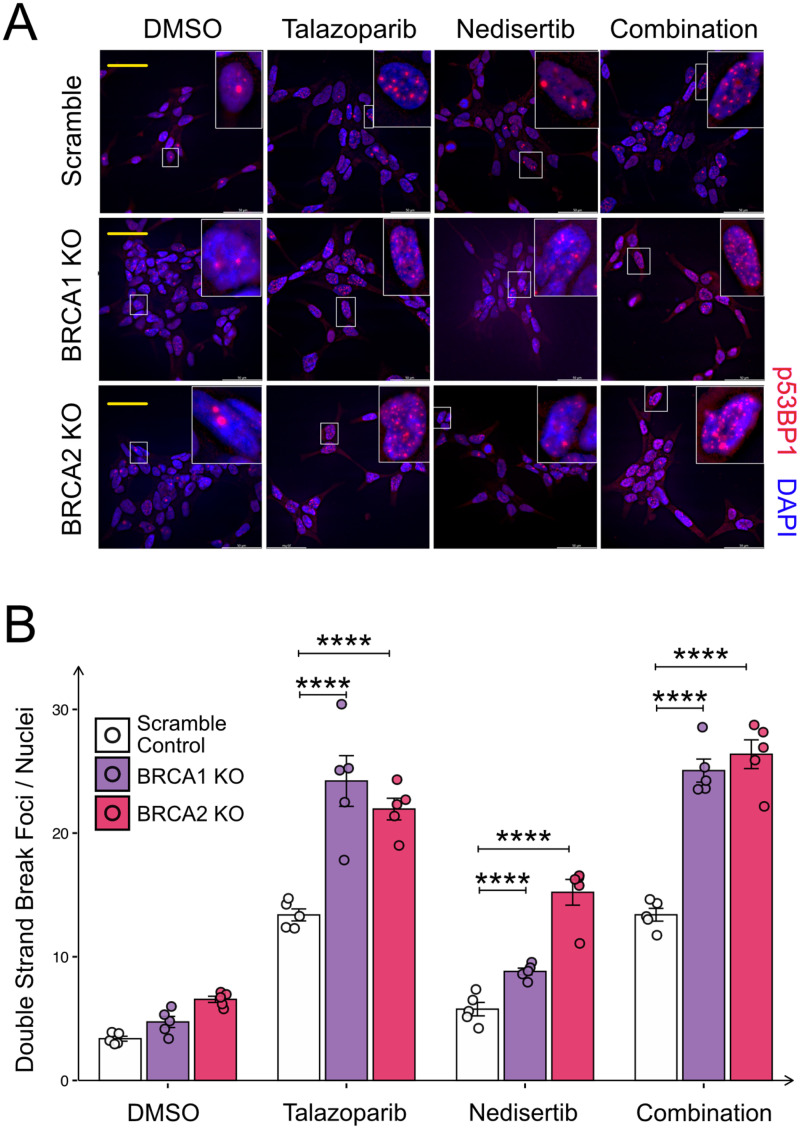
Co-Targeting PARP and DNA-PK Increases DSBs in BRCA1/2 Deficient Cells. (A) Representative Immunofluorescence Images Highlighting the Presence of DNA Double- Strand Breaks (DSB), Marked by red-Staining of p53BP1 and the Nucleus Marked by Blue Staining via DAPI. Scale bar: 50μm. (B) Quantification of DSB per Cell Nuclei in BRCA1/2 Knockout LNCaP Cells Compared to the Scramble Control for Five Independent Experiments. Graphs Represent Mean ± SEM. p-Values: p < 0.0001 (****).

53BP1 is a damage response factor and functions at DSB making it a reliable marker for DSB. To assess the influence of talazoparib, nedisertib and their combination on the accumulation of DSB we conducted an immunofluorescence staining to quantify 53BP1 foci in LNCaP scramble, BRCA1 KO and BRCA2 KO cells. The cells were treated for 48 h with 8 nM talazoparib, 1000 nM nedisertib and the combination. We observed a significant increase in DSB/cell between the KO's of BRCA 1 and 2 compared to scramble after treatment of talazoparib, nedisertib and the combination treatment (Figure 3A). The combination treatment resulted in the highest number of DSB/foci that were quantified among the treatment conditions (Figure 3B).

### DNA-PK Amplification Predicts Poor Prognosis, While BRCA1/2 Loss Enhances DNA-PK Activity *in Vitro*

**Figure 4.**
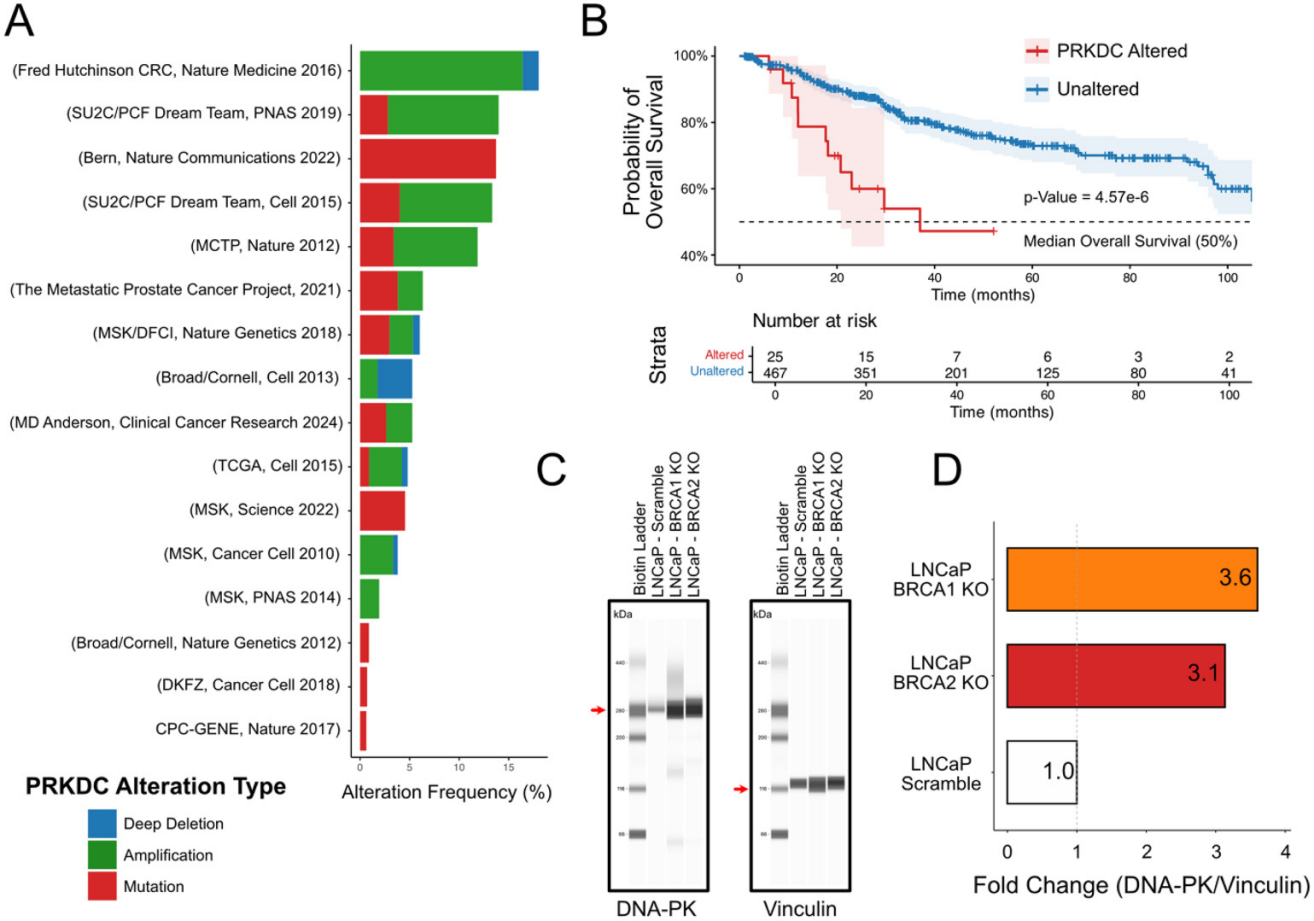
DNA-PK Amplification Predicts Poor Prognosis; BRCA1/2 Loss Elevates DNA-PK Activity. (A) Frequency of PRKDC (DNA-PK) Alterations, Including Mutations (red), Amplification (Green) and Deep Deletions (Blue), Across the CBioPortal Database for Prostate Cancer. (B) Kaplan–Meier Analysis of Overall Survival Comparing Patients with PRKDC Alterations (red) to Those with Unaltered PRKDC (Blue), Demonstrating Significantly Reduced Survival in the Altered Group. n = 25 in PRKDC Altered Group; n = 467 in the Unaltered Group. (C) Representative Simple Western Lane View of Untreated Scramble LNCaP Cells or BRCA1/2 Knockout LNCaP Cells Probed with DNA-PK or Vinculin Antibodies. Red Arrows Demonstrate the Band of Interest. (D) Densitometric Quantification of DNA-PK Normalized to Vinculin. Values are Represented as a Fold Change Compared to the Scramble Control.

We hypothesized that PCa tumors acquire resistance to PARP inhibition through NHEJ mediated by DNA-PK. To test whether DNA-PK expression correlates with overall survival (OS), we reanalyzed genomic and clinical data from 12 361 samples across 1893 patients using publicly available cBioPortal datasets. Our analysis showed that most DNA-PK (PRKDC) alterations were gene amplifications, which inversely correlated with BRCA1/2 alterations, predominantly deep deletions or inactivating mutations (Figure 4A). Patients with PRKDC alterations showed a significantly shorter overall survival, compared to those without alterations, at the time point corresponding to 50% overall mortality within the cohort (Figure 4B). Simple Western analysis revealed that BRCA1/2 knockout LNCaP cells exhibit increased DNA-PK expression even in the absence of treatment (Figure 4C and D). This suggests that loss of BRCA1/2 predisposes cells to elevated DNA damage, leading to compensatory upregulation of DNA-PK to facilitate repair through NHEJ. Consequently, dual inhibition of PARP and DNA-PK exerts a synergistic effect by promoting synthetic lethality in this subset of tumor cells.

## Discussion

Highly aggressive PCa is often linked to defects in DNA damage repair genes, particularly *BRCA1* and *BRCA2*.^1,2,6–10^ *BRCA* genes help maintain genomic stability by repairing DSBs through homologous recombination (HR), which uses the second undamaged DNA strand as a template for accurate repair.^4^ While PARPi have improved treatment outcomes for patients with BRCA mutated PCa, tumor cells commonly develop resistance over time.^14,15^

**Figure 5.**
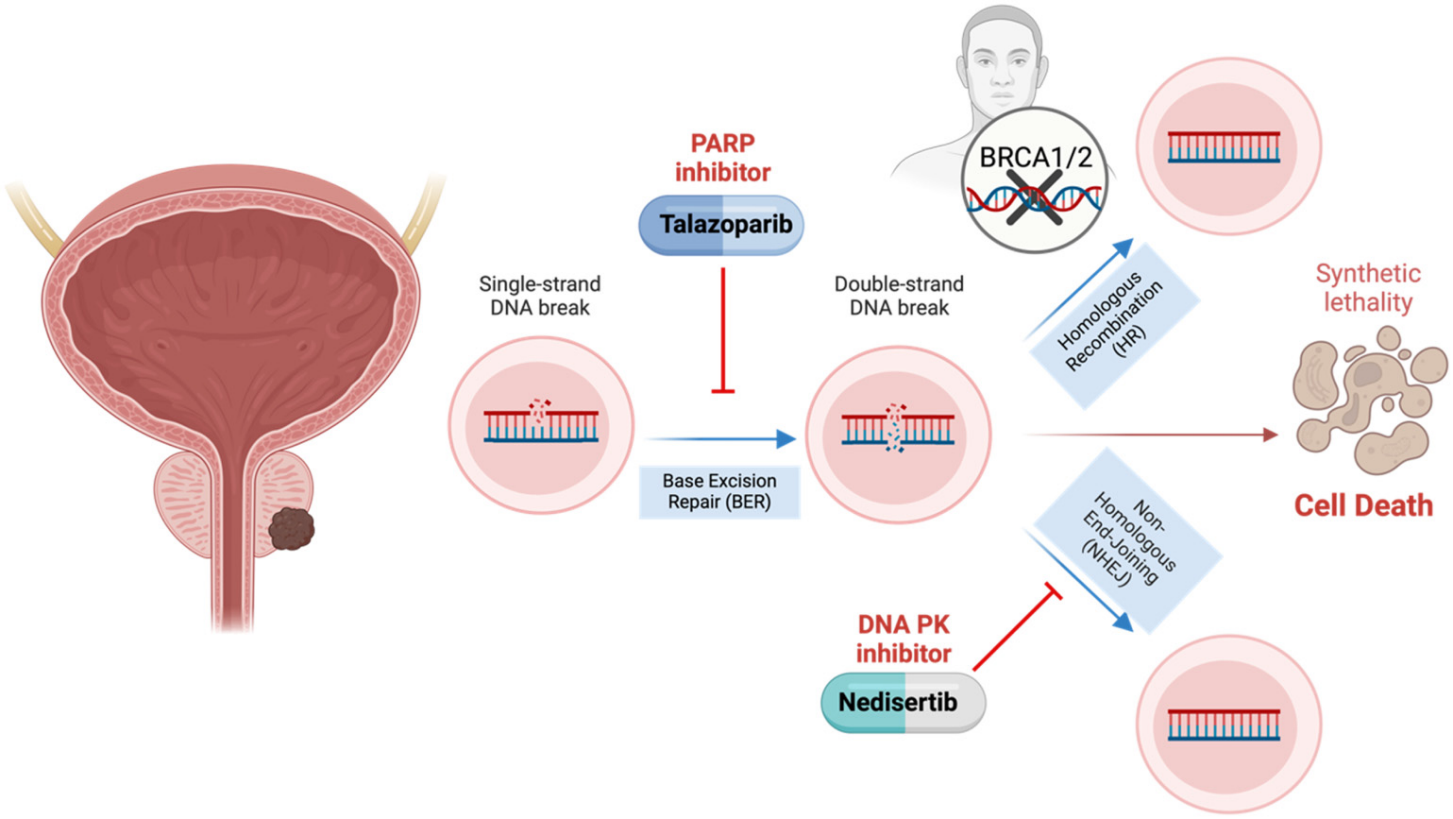
Schematic Representation of the Study's Objective, Focusing on the Therapeutic Potential of DNA-PK Inhibitors Combined with PARP Inhibitors in Prostate Cancer with *BRCA1* and *BRCA2* Gene Mutations. Created in BioRender (Mazumdar).^46^

In our experiments, treatment with the combination of nedisertib and talazoparib for 48 h led to a marked accumulation of DSBs in BRCA1/2-deficient cells, with treated nuclei exhibiting an average of over 20 DSBs per cell. The G2/M DNA damage checkpoint is thought to tolerate up to approximately 10-20 DSBs before triggering arrest.^45^ At higher levels of damage, cells may either go into prolonged cell cycle arrest or premature mitotic entry. BRCA-deficient cells, impaired in homologous recombination, are unable to efficiently repair such damage, especially under continued inhibition of alternative repair pathways via nedisertib and talazoparib. This unresolved genomic instability may lead to mitotic catastrophe or necrotic cell death, consistent with the non-apoptotic phenotype observed in our study (Figure 5).

PARP enzymes are DNA damage sensors that bind DNA single-strand breaks, resulting in the initiation of base excision repair.^47^ PARPi block the enzymatic activity of PARP and promote its trapping at sites of DNA damage, ultimately leading to cell death.^48,49^ *In vitro* data indicate that the PARP trapping mechanism seems more cytotoxic than enzymatic inhibition.^50^ Compared with other PARPi, talazoparib displays elevated PARP-trapping potential.^49^

PARPi are frequently combined with androgen receptor pathway inhibitors (ARPI) as the combination improves outcomes in patients with intact HR.^51^ The current guideline of the American Society of Clinical Oncology recommends PARPi in metastatic castration-resistant PCa in addition to ARPI if patients are ARPI-naïve, whereas PARPi monotherapy is reserved for patients with prior ARPI exposure and HR mutation.^52^ Androgen deprivation remains the cornerstone of systemic treatment in advanced PCa.^53^ However, the optimal sequencing and combination strategies are still unclear. As next-generation sequencing and liquid biopsies becomes more accessible, earlier identification of patients with HR deficiency will facilitate more personalized treatment planning.^54^

Although PARPi are the recommended treatment in HR deficient patients, their long-term therapeutic effectiveness is limited since resistance to them often arises within months of starting medication.^16^ Various pathways to develop resistance to PARPi treatment have been described, leading to the restoration of DNA repair and tumor growth.^55^ One possible escape mechanism involves the activation of the NHEJ repair pathway. In contrast to HR, NHEJ repairs DSBs without the need for a template, often resulting in changes to the DNA sequence.^56^ NEHJ is responsible for repairing the majority of DSBs caused by ionizing radiation in cancer cells.^56^ Therefore, alteration of the NHEJ has become a target for anti-tumor therapy.^57^ Nedisertib has shown to be an effective inhibitor of DNA-PK resulting in suppression of NHEJ. Inhibition of DNA-PK was shown to resensitize BRCA2 deficient, PARPi resistant mammary tumor cells.^58^ In addition to NHEJ, microhomology-mediated end joining (MMEJ) is another important alternative DSB repair pathway that may facilitate resistance to PARPi.^59^ MMEJ is an error-prone mechanism that uses short regions of microhomology to align DNA ends, often resulting in deletions at the repair site. Notably, MMEJ is frequently upregulated in HR-deficient tumors, including those harboring BRCA1/2 mutations.^59,60^

DNA-PK has become a target for various antitumor therapies as it not only plays a central role in DNA repair pathways but is also active in several cellular processes, including transcriptional regulation, cell cycle progression and telomere maintenance.^61^ In PCa, the upregulation of DNA PK is associated with metastatic disease, recurrence and worse survival.^62^ Furthermore, DNA-PK has shown to be a co-activator of the androgen receptor, which plays a key role in PCa progression.^62^ PARPi monotherapy shows antitumor activity in HR-deficient patients harboring further mutations in PALB2, BRIP1, or FANCA, whereas individuals with ATM or cyclin-dependent kinase 12 (CDK12) mutations appear to derive little benefit. Especially, biallelic inactivation of CDK12 has potential as a biomarker for treatment response. Because it plays a role in controlling genomic stability, it could define an immuno-responsive phenotype.^63^

The combination of DNA-PK and PARP inhibitors, as demonstrated in xenograft and patient-derived xenograft models, increased genomic instability, growth inhibition, and apoptosis in breast cancer, and reduced the growth of hepatocellular carcinoma.^64,65^ Nedisertib is currently being investigated in multiple clinical trials as a radiosensitizer across various tumor types.^66^ One study currently recruiting is conducted with patients suffering from castrate-resistant PCa undergoing treatment with radium-223 (NCT04071236). Radium-223 is an alpha emitter that mimics Calcium and is, therefore, incorporated into bone metastasis. It has demonstrated significant survival benefits.^67^ Our study results support the use of nedisertib in PCa and suggest possible additional benefits in patients with BRCA mutations. Furthermore, with the advancement in tumor-guided radioligand therapy targeting prostate-specific membrane antigen (PSMA), nedisertib offers the potential to further enhance the cytotoxic effects, which potentially is especially effective in BRCA-mutated patients.

This study has limitations. First, although CRISPR-Cas9-mediated knockouts successfully disrupted BRCA1 and BRCA2 protein expression, off-target effects of genome editing cannot be completely excluded. Additionally, since the therapeutic efficacy and tolerability of combined PARP and DNA-PK inhibition were not evaluated *in vivo*, the translational relevance of the findings is limited. Future studies are planned to validate these results.

## Conclusion

In conclusion, we successfully established BRCA1 and BRCA2 knockout PCa cell models using CRISPR-Cas9. We demonstrated that interfering with the non-homologues DNA double strand repair using DNA PK inhibitors seems to be a promising approach in BRCA-deficient cells (Figure 5). When combining DNA PK and PARP inhibitors, we observed additive effects on cell proliferation, more frequent DNA damage, and elevated necrosis. Based on these findings, future studies have to assess the efficacy and safety of this combination therapy *in vivo* to explore this possible treatment strategy for patients with BRCA-mutated PCa.

## Supplemental Material

sj-docx-1-tct-10.1177_15330338251394948 - Supplemental material for Combined DNA-PK and PARP Inhibition as a Therapeutic Strategy in BRCA-Mutated Prostate Cancer: An in Vitro Pilot Study Supplemental material, sj-docx-1-tct-10.1177_15330338251394948 for Combined DNA-PK and PARP Inhibition as a Therapeutic Strategy in BRCA-Mutated Prostate Cancer: An in Vitro Pilot Study by Thomas Paul Scherer, MD, MPH, Souzan Salemi, PhD, Valentin Baumgartner, PhD, Dominik Enderlin, MD, Alekhya Mazumdar, PhD, and Daniel Eberli, MD, PhD in Technology in Cancer Research & Treatment

## Abbreviations

ANOVA: analysis of variance
AR: androgen receptor
ARPI: androgen receptor pathway inhibitor
BCA: bicinchoninic acid
BSA: bovine serum albumin
BRCA1: breast cancer gene 1
BRCA2: breast cancer gene 2
CDK12: cyclin-dependent kinase 12
CRISPR: clustered regularly interspaced short palindromic repeats
Cy3: cyanine 3
DAPI: 4’,6-diamidino-2-phenylindole
DMEM: dulbecco’s modified eagle medium
DNA-PK: DNA-dependent protein kinase
DSB: double-strand break
EDTA: ethylenediaminetetraacetic acid
FACS: fluorescence-activated cell sorting
FBS: fetal bovine serum growth supplement for cell culture media
FITC: fluorescein isothiocyanate
gRNA: guide RNA
HEK: human embryonic kidney
HR: homologous recombination
KO: knockout
LNCaP: lymphnode cancer of the prostate (cell line)
MMEJ: microhomology-mediated end joining
NHEJ: non-homologous end joining
OS: overall survival
PARP: Poly (ADP-Ribose) Polymerase
PARPi: PARP inhibitor
PBS: phosphate-buffered saline
PEI: polyethylenimine
PI: propidium iodide
PRKDC: Protein Kinase, DNA-Activated, Catalytic Subunit
PSMA: prostate-specific membrane antigen
RT: room temperature
SEM: standard error of the mean
sgRNA: single-guide RNA
SSB: single-strand break
WES: western blot
