# Supplementary material for: Combined DNA-PK and PARP Inhibition as a Therapeutic Strategy in BRCA-Mutated Prostate Cancer: An in Vitro Pilot Study
Source: Technol Cancer Res Treat. 2025 Nov 14;24:15330338251394948. doi: 10.1177/15330338251394948 (PMC12618835; doi:10.1177/15330338251394948)
Supplement: sj-docx-1-tct-10.1177_15330338251394948 - Supplemental material for Combined DNA-PK and PARP Inhibition as a Therapeutic Strategy in BRCA-Mutated Prostate Cancer: An in Vitro Pilot Study [file sj-docx-1-tct-10.1177_15330338251394948.docx]

| **Name** | **Sequence (5‘-3‘)** |
| --- | --- |
| **sg_Scramble (1)** | GTAGCGAACGTGTCCGGCC |
|  |  |
| **sg_BRCA1 (1)** | TGGTCACACTTTGTGGAGAC |
| **sg_BRCA1 (2)** | GGTTTCTGTAGCCCATACTT |
|  |  |
| **sg_BRCA2 (1)** | GTCTACCTGACCAATCGATG |
| **sg_BRCA2 (2)** | AGCACAGTAGAACTAAGGGT |
| **sg_BRCA2 (3)** | GCTCGCTGGTATACCAAACTTGG |

**Supplemental Table 1.**

Guide RNAs for CRISPR-Cas9 -mediated deletion of BRCA1 and BRCA2

**Supplemental Figure 1**


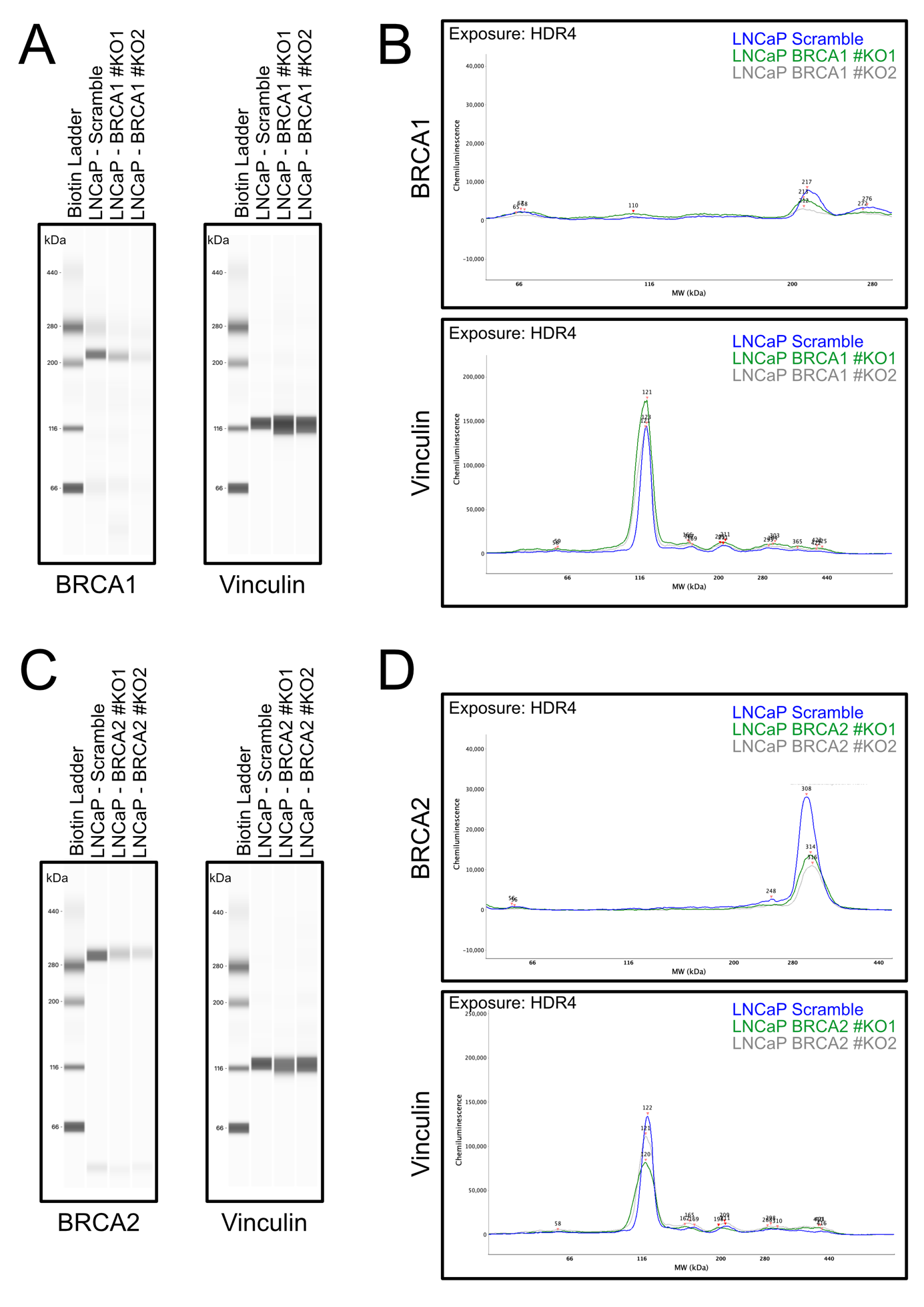


Supplemental Figure 1: (A) Representative full lane view of scramble control LNCaP cells or BRCA1 KO LNCaP cells after Simple Western Analysis. (B) Graph view of BRCA1 staining as analysed by the Simple Western software. Data are displayed as an electropherogram, with migration distance on the X-axis and chemiluminescence intensity on the Y-axis. Quantification is obtained from the area under the curve. (C) Representative full lane view of scramble control LNCaP cells or BRCA2 KO LNCaP cells after Simple Western Analysis. (D) Respective graph view of BRCA2 staining as analysed by the Simple Western software. The same Vinculin quantification was used as the housekeeping control for the scramble condition in both BRCA1 and BRCA2 staining.

**Supplemental Figure 2**

**
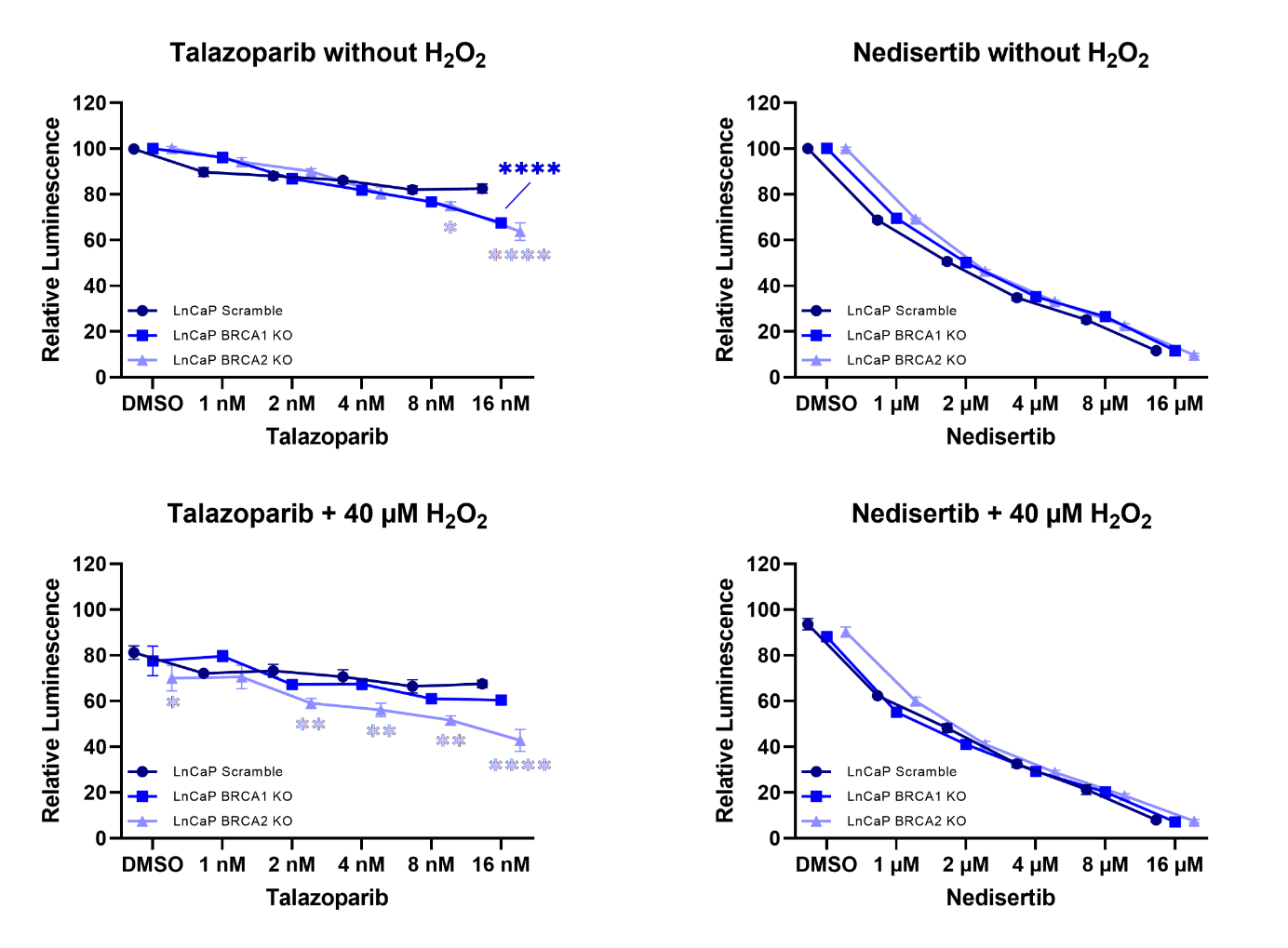
**

Supplemental Figure 2: Dose–response curves of LNCaP cells with or without BRCA knockout following treatment with talazoparib or nedisertib.

LNCaP cells (Scramble control, BRCA1 KO, and BRCA2 KO) were treated with increasing concentrations of talazoparib (left) or nedisertib (right) for 48h. Cell viability was assessed using the CellTiter-Glo 2.0 assay. Data represent the mean ± SD of three independent experiments. p-Value < 0.001 (****).
